# Supplementary material for: Global nonlinear approach for mapping parameters of neural mass models
Source: PLoS Comput Biol. 2023 Mar 24;19(3):e1010985. doi: 10.1371/journal.pcbi.1010985 (PMC10075456; doi:10.1371/journal.pcbi.1010985)
Supplement: S1 Fig — (PDF) [file pcbi.1010985.s001.pdf]

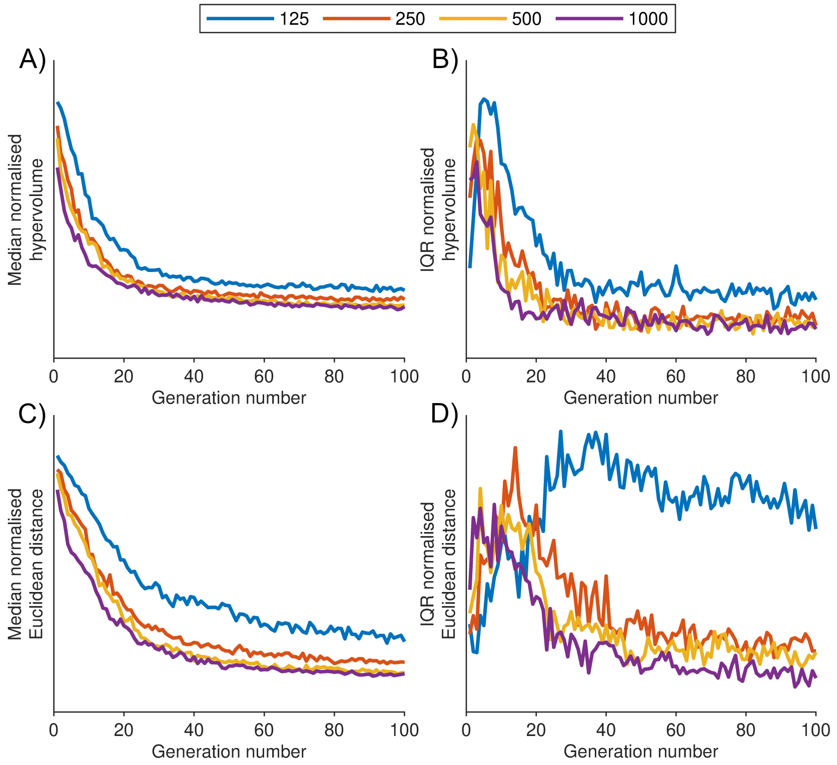

S1 Fig. Normalised convergence metrics of optimisation for different population sizes. Colours as per legend. This is given for fitting to an example data subject (subject 4) using the MOEA20 approach. A) gives the median hypervolume indicator (obtained from the full non-dominated set) over generation number. B) gives the interquartile range (IQR) over generation number. C) gives the median value of the point with the best Euclidean distance from the origin (see Methods) over generation number. D) gives the median IQR of the point with the best Euclidean distance from the origin over generation number.
