# Supplementary material for: Global nonlinear approach for mapping parameters of neural mass models
Source: PLoS Comput Biol. 2023 Mar 24;19(3):e1010985. doi: 10.1371/journal.pcbi.1010985 (PMC10075456; doi:10.1371/journal.pcbi.1010985)
Supplement: S2 Fig — (PDF) [file pcbi.1010985.s002.pdf]

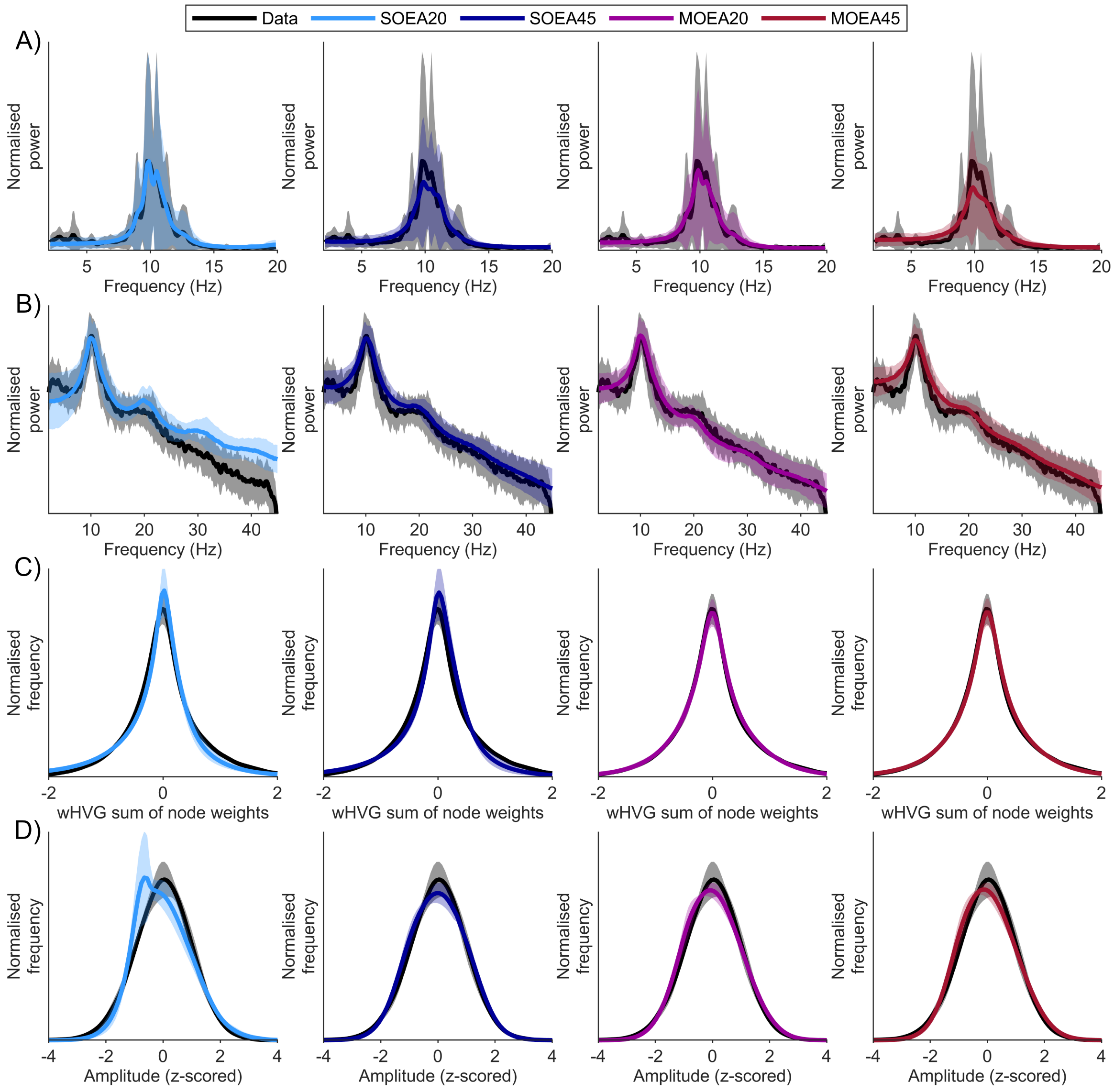


**S2 Fig. PSD, wHVG and amplitude distributions derived from optimal model simulations for all control subjects, using each algorithm.** Optimal refers to the smallest Euclidean distance from the origin in objective space. Mean values are denoted as lines, with the shaded region representing the standard deviation over subjects (black denotes data, colours as per legend). A) PSD in the 2-20Hz range, B) PSD log-transformed in the 2-45Hz range, C) wHVG degree distribution and D) amplitude distribution. For the wHVG and amplitude distributions, these signals show a density approximation calculated from 100 equally sized bins (see Methods).
