# Supplementary material for: Global nonlinear approach for mapping parameters of neural mass models
Source: PLoS Comput Biol. 2023 Mar 24;19(3):e1010985. doi: 10.1371/journal.pcbi.1010985 (PMC10075456; doi:10.1371/journal.pcbi.1010985)
Supplement: S3 Fig — (PDF) [file pcbi.1010985.s003.pdf]

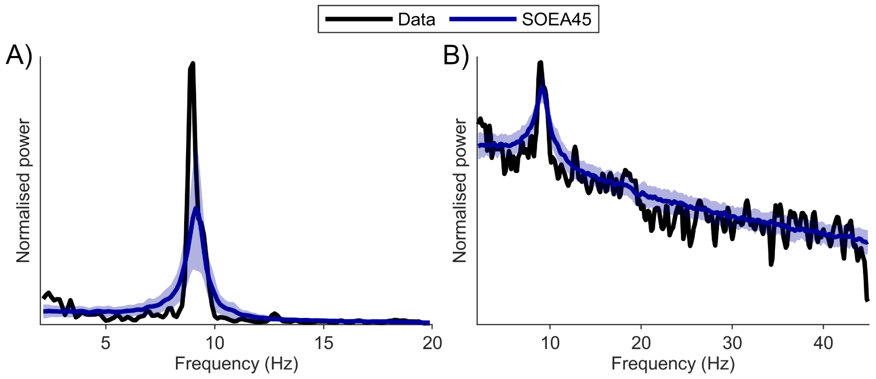

S3 Fig. Example of the SOEA45 missing the dominant alpha peak in favour of better fitting the 2-45Hz range. A) shows the data PSD in the 2-20Hz range for this subject. The optimal model fits to data using the SOEA45 are shown by the blue line (mean value) and blue shaded region (standard deviation across 100 repeats). Optimal refers to the smallest Euclidean distance from the origin in objective space. B) shows the PSD in the log-transformed 2-45Hz range. Colours as per legend.
