# Supplementary material for: Global nonlinear approach for mapping parameters of neural mass models
Source: PLoS Comput Biol. 2023 Mar 24;19(3):e1010985. doi: 10.1371/journal.pcbi.1010985 (PMC10075456; doi:10.1371/journal.pcbi.1010985)
Supplement: S4 Fig — (PDF) [file pcbi.1010985.s004.pdf]

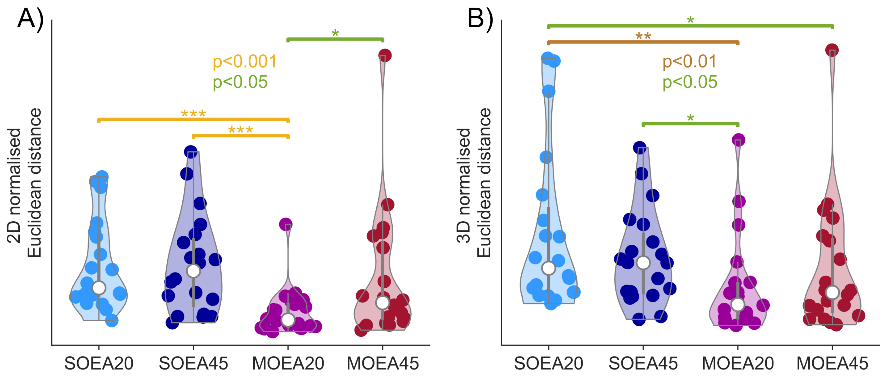


**S4 Fig. Comparison of normalised objective scores across the four algorithms.** A) shows the mean Euclidean distance of the normalised objective scores in 2-dimensional space (PSD 2-20Hz and wHVG distribution) for each algorithm. B) shows the mean Euclidean distance of the normalised objective scores in 3-dimensional space (PSD 2-20Hz, log-transformed PSD 2-45Hz and wHVG distribution) for each algorithm. Mean values were obtained across the 100 repeats of the algorithms on each subject. The p-values indicated were obtained from a Mann-Whitney U test. Scores were normalised between 0 and 1 before the Euclidean distance was calculated.
