# Supplementary material for: Global nonlinear approach for mapping parameters of neural mass models
Source: PLoS Comput Biol. 2023 Mar 24;19(3):e1010985. doi: 10.1371/journal.pcbi.1010985 (PMC10075456; doi:10.1371/journal.pcbi.1010985)
Supplement: S5 Fig — (PDF) [file pcbi.1010985.s005.pdf]

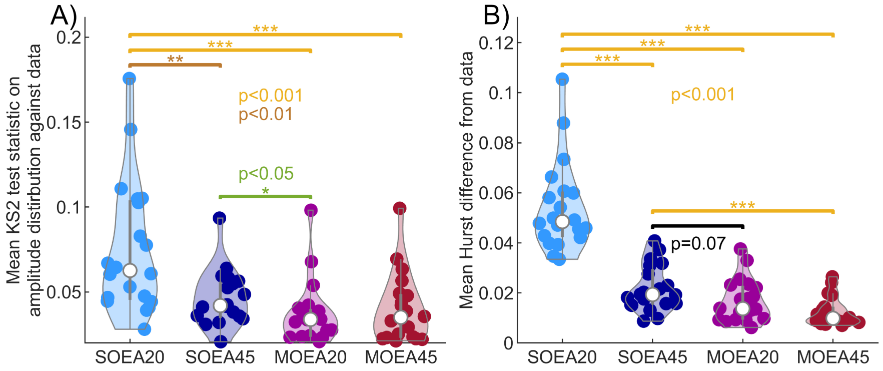

S5 Fig. Distributions of amplitudes and Hurst exponents calculated from optimal model simulations. A) shows the mean two-sided Kolmogorov-Smirnov (KS2) test statistic between the amplitude distribution of optimal model simulations and data. Optimal refers to the smallest Euclidean distance from the origin in objective space. B) shows the mean difference between the Hurst exponent of optimal model simulations and data. P-values were obtained from a Mann-Whitney U test.
