# Supplementary material for: Global nonlinear approach for mapping parameters of neural mass models
Source: PLoS Comput Biol. 2023 Mar 24;19(3):e1010985. doi: 10.1371/journal.pcbi.1010985 (PMC10075456; doi:10.1371/journal.pcbi.1010985)
Supplement: S8 Fig — (PDF) [file pcbi.1010985.s008.pdf]

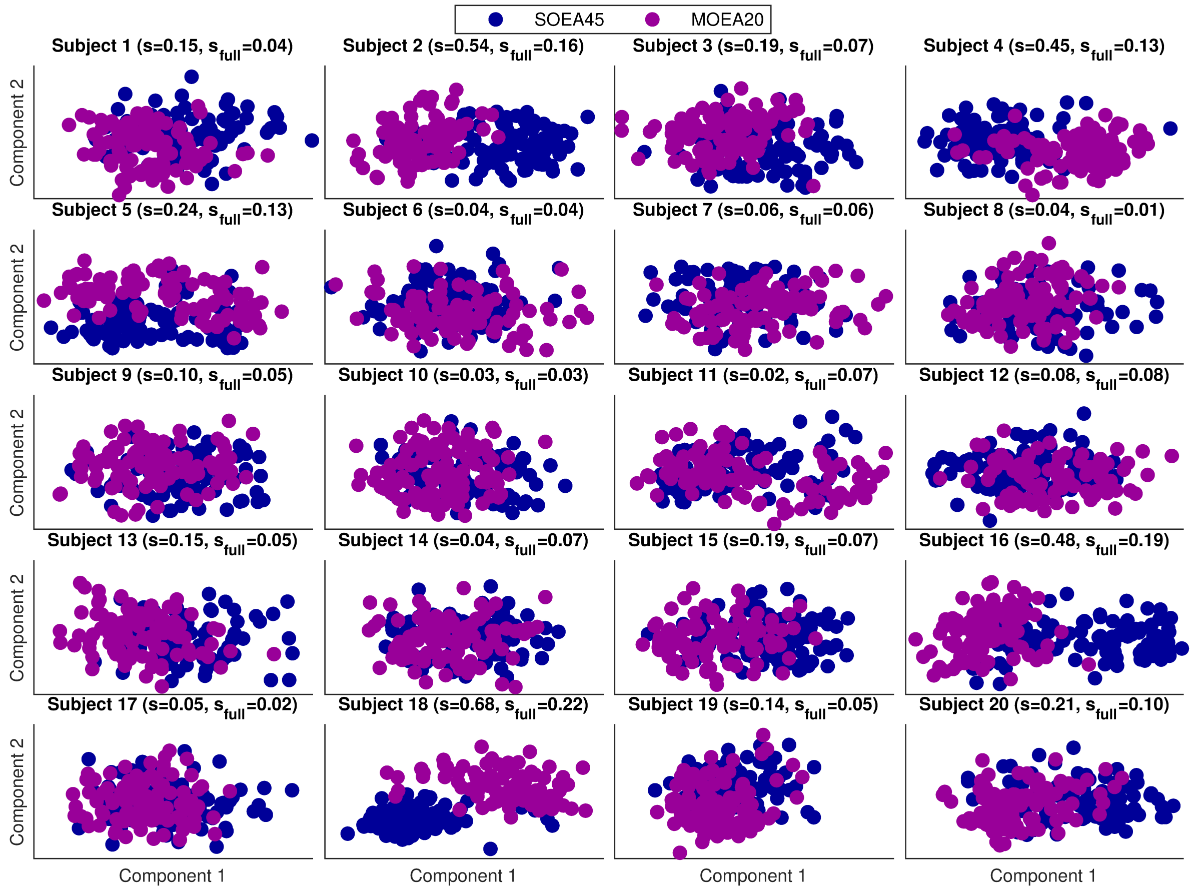

S8 Fig. SOEA45 and MOEA20 parameters in 2-d space after a multi-dimensional scaling was applied to the parameters in the full space. This is shown for optimal parameter sets from all control subjects. Optimal refers to the smallest Euclidean distance from the origin in objective space. The silhouette score in the reduced 2-d space and the full space is provided in each subplot title.
