# Supplementary material for: Global nonlinear approach for mapping parameters of neural mass models
Source: PLoS Comput Biol. 2023 Mar 24;19(3):e1010985. doi: 10.1371/journal.pcbi.1010985 (PMC10075456; doi:10.1371/journal.pcbi.1010985)
Supplement: S12 Fig — (PDF) [file pcbi.1010985.s012.pdf]

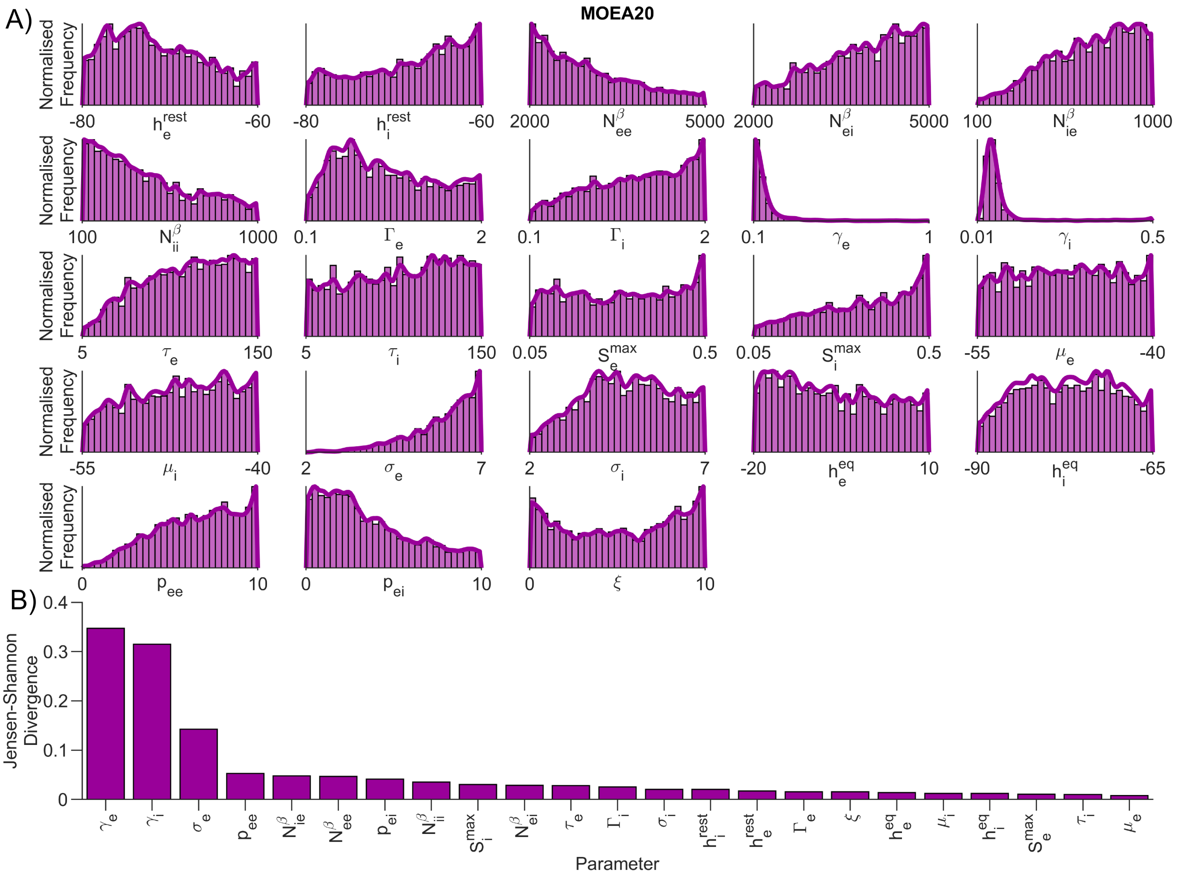

S12 Fig. Parameter distributions recovered from resting EEG of all control subjects using the MOEA20 approach. A) shows the univariate parameter distributions for all parameters in the model. The parameter bounds are set to those used in the optimisation (see Table 1). B) shows the corresponding JSD of the parameters from a uniform distribution.
