# Supplementary material for: Global nonlinear approach for mapping parameters of neural mass models
Source: PLoS Comput Biol. 2023 Mar 24;19(3):e1010985. doi: 10.1371/journal.pcbi.1010985 (PMC10075456; doi:10.1371/journal.pcbi.1010985)
Supplement: S14 Fig — (PDF) [file pcbi.1010985.s014.pdf]

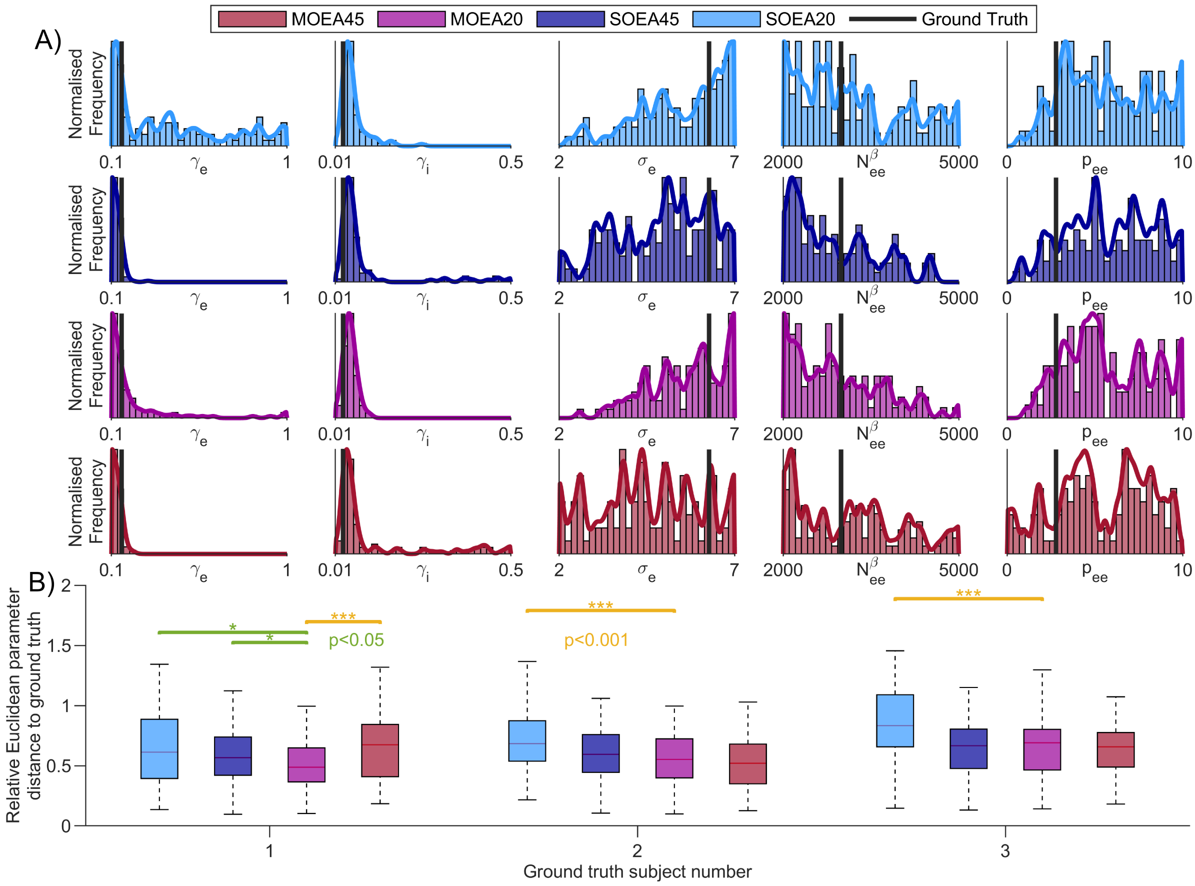


**S14 Fig. Parameter distributions recovered from simulated subjects.** A) shows the univariate parameter distributions for the 5 most identifiable parameters for a single simulated subject. The ground truth value used to simulate the dynamics for this subject is given by the black line. The parameter bounds are set to those used in the optimisation (see Table 1). B) shows the relative Euclidean parameter distance to the ground truth across the 5 parameters, for 3 different ground truths. P-values were obtained from a Mann-Whitney U test, with Bonferroni correction.
