# Supplementary material for: Global nonlinear approach for mapping parameters of neural mass models
Source: PLoS Comput Biol. 2023 Mar 24;19(3):e1010985. doi: 10.1371/journal.pcbi.1010985 (PMC10075456; doi:10.1371/journal.pcbi.1010985)
Supplement: S15 Fig — (PDF) [file pcbi.1010985.s015.pdf]

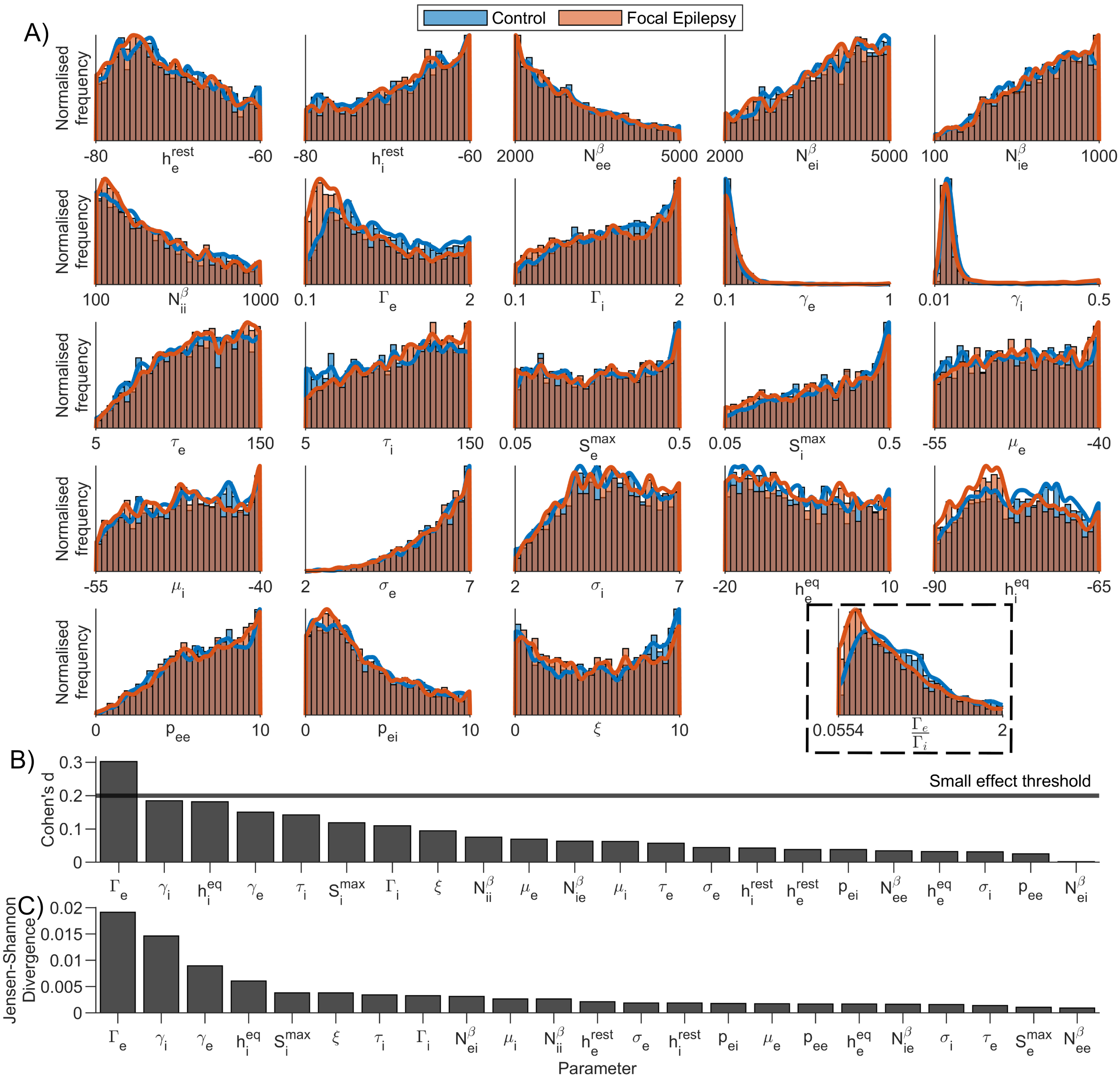

S15 Fig. Distributions of all parameters recovered from FE and control EEG data. A) shows the univariate distributions of each parameter. The parameter bounds are set to those used in the optimisation (see Table 1). Note that $\frac{\Gamma_e}{\Gamma_i}$ is boxed because it is not a model parameter, but instead explicitly signifies the difference in the excitation/inhibition ratio between the cohorts. B) shows the ordered Cohen's d effect size comparing the means of the control and FE recovered parameter distributions. C) shows the ordered JSD comparing the recovered control and FE parameter distributions. Colours as per legend.
