# Supplementary material for: Global nonlinear approach for mapping parameters of neural mass models
Source: PLoS Comput Biol. 2023 Mar 24;19(3):e1010985. doi: 10.1371/journal.pcbi.1010985 (PMC10075456; doi:10.1371/journal.pcbi.1010985)
Supplement: S16 Fig — (PDF) [file pcbi.1010985.s016.pdf]

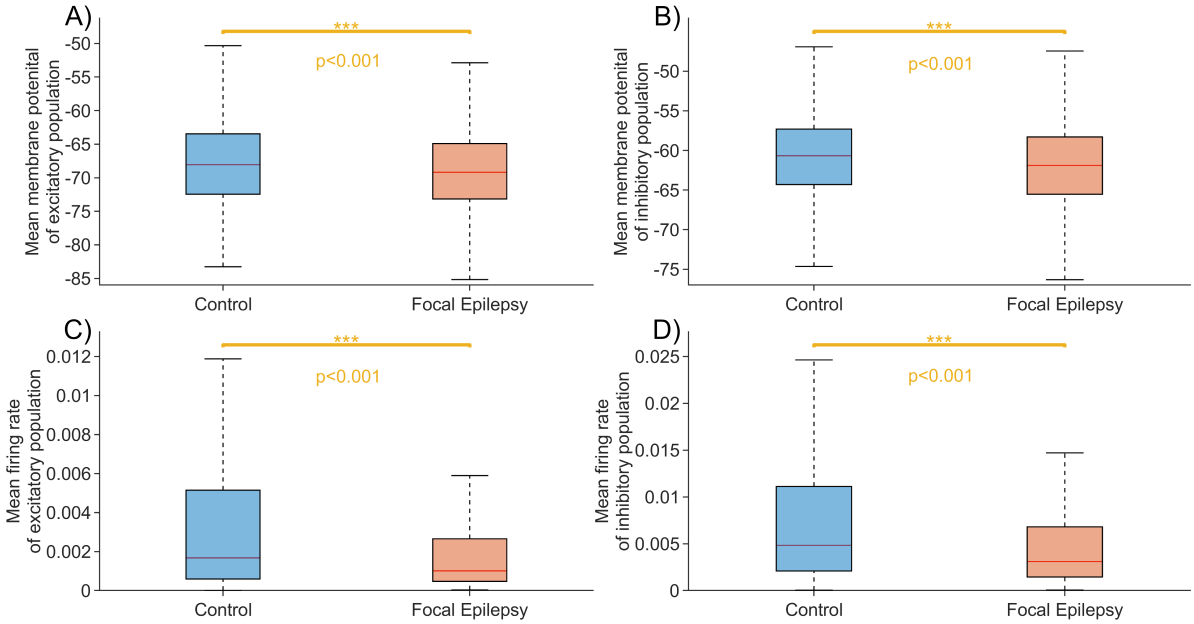

S16 Fig. Comparison of mean membrane potentials and firing rates of excitatory and inhibitory populations at optimal parameters. Optimal refers to the smallest Euclidean distance from the origin in objective space. A) and B) show the control and FE mean membrane potential from the excitatory and inhibitory populations, respectively. C) and D) show the control and FE mean firing rates from the excitatory and inhibitory populations, respectively. Boxplots comprise simulations at 100 optimal parameter values recovered from all subjects in each cohort. The p-values indicated were obtained from a Mann-Whitney U test.
