# Supplementary material for: Global nonlinear approach for mapping parameters of neural mass models
Source: PLoS Comput Biol. 2023 Mar 24;19(3):e1010985. doi: 10.1371/journal.pcbi.1010985 (PMC10075456; doi:10.1371/journal.pcbi.1010985)
Supplement: Attachment — Submitted filename: Dunstan_et_al_NMM_parameter_fitting_2022.pdf [file pcbi.1010985.s018.pdf]

Global nonlinear approach for mapping parameters of neural mass models
Dominic M. Dunstan, Mark P. Richardson, Eugenio Abela, Ozgur E. Akman, Marc Goodfellow

Overview
The authors present a global optimisation technique for fitting neural mass models to EEG data. They show that using different objective functions in the optimisation results in different model fits. They then apply their method to identify differences in the parameter values for health controls versus patients with focal epilepsy. The work is timely and relevant, and the paper is well written. However, I have a few concerns with how the work and results are explained/presented.

- The results show how different objective functions recover different parameter sets, yet the authors claim that different objective functions recover different dynamics. I am aware that changing parameters changes the model behaviour. However, such changes in behaviour are often minor. If the authors wish to state that the different objective functions recover different dynamics, they need to substantiate these claims with additional simulation (could be given in an appendix).

- I am not really sure what the take home message is in terms of the comparison of the different objective functions. Is one better than the others? Does it depend on what properties of the EEG signal you are interested in? Or perhaps, one should be combining all four methods and using only the overlapping regions of parameter space?

- The authors use the sign of the dominant eigenvalue as a measure of whether the dynamics are linear (negative real part) or non-linear (positive real part). This is incorrect. Non-linear systems can have stable fixed points and, as such, eigenvalues which all have negative real parts. Take a stable spiral, for example, variables oscillate with a decaying amplitude as we approach the fixed point. This is not linear behaviour.

- The results for spike-wave dynamics are interesting but seem somewhat like an afterthought. There are many more interesting questions that could be answered here. For example, how similar/different are the parameters values for SWDs to those for regular oscillations in patients with epilepsy? Ideally, the system should live in a region of parameter space where both types of behaviour are possible. Then noise or small variations to the parameter values would lead to switches between the two behaviours. I appreciate that such an exploration maybe outside the scope of this paper, but the authors should at least mention how similar/different this parameter set is to the parameter sets found in the previous parts.

Outside of these concerns, I have a question about the results related to the multi-dimensional scaling and the silhouette scores. Can you tell which parameters contribute most to the differences in the 2-dimensional components? If so, is there a reason for certain parameters contributing more or less? Can we gain any insight into why this would be, i.e. what is the significance of these parameters?

Minor comments/corrections

Throughout

- Abbreviations – it would be helpful for the reader if the abbreviations are restated when they are used for the first time in a while. I found myself constantly flicking back and forth trying to figure out what certain abbreviation were. For example, when SWD is used on pg 21 after not being used since pg 6. Even better, you could include a table with all of the abbreviations.
- Axis labels and ranges/values
  - Many figures do show the range of the values on the y-axis, some also do not have the range for the x-axis
  - Figure 5 – Am I correct in saying that the plots shown in panel a and b are histograms showing the frequency of occurrence of each parameter value? If so, the parameter name should be on the x-axis, with range of the parameter values also indicated on this axis. The y-axis should have a label such as frequency and the range of this value should be indicated on each panel (can be on the leftmost panel if the range is the same for all panels)
  - Figure 7 and Figure 10 (a) – Same comment as for Figure 5

Abstract

- “comprise many parameters” I don’t think comprise is the correct word to use here, and if it is it should be “comprise of many parameters” (used again in introduction on line 28)
- “constrain model output” -> “constrain the model output”
- “MOEA can be used” -> “MOEA could used”

Author summary

- Remove “The” before “EEG”

Methods

- If you put a backslash before the exp in your equations, it will be written in regular font rather than italics
- Remove “that” after’ “incorporate” on line 137

Results

- Figure 1 – it would be useful to see the time course of the zoomed in inset with the 5 points marked on it. Perhaps it could be included as part of/above panel d
- Figure 1 caption: “shows 5 consecutive time points” -> “shows the amplitude of 5 consecutive time points”
- Line 267, “It” should be “it” (not at the start of sentence)
- Figure 7 and associated results - are the difference in JSD significant? I encourage the authors to perform hypothesis tests to confirm that they are.
