# Supplementary material for: Global nonlinear approach for mapping parameters of neural mass models
Source: PLoS Comput Biol. 2023 Mar 24;19(3):e1010985. doi: 10.1371/journal.pcbi.1010985 (PMC10075456; doi:10.1371/journal.pcbi.1010985)
Supplement: Attachment — Submitted filename: Reponse to review comments.pdf [file pcbi.1010985.s019.pdf]

Department of Mathematics and Statistics
Living Systems Institute
University of Exeter
Stocker Road
Exeter
EX4 4QD, UK
Email: d.dunstan@exeter.ac.uk

31th January 2023. RE: resubmission of revised manuscript: Global nonlinear approach for mapping parameters of neural mass models.

Dear Editors,

Thank you for considering our manuscript for publication at PLOS Computational Biology. We would additionally like to thank the reviewers for their constructive comments on the manuscript.

Included below is a list of the responses to the reviewers' comments and a description of the corresponding changes to the manuscript that have been made. In the submission is an updated manuscript and supporting information both with and without these changes highlighted.

Should you need any further information, please be in touch.

Yours sincerely,

Dominic Dunstan, Mathematics and Statistics and LSI, University of Exeter

General comments

Have the authors made all data and (if applicable) computational code underlying the findings in their manuscript fully available?

Computation code to run algorithms and recreate the main figures in the manuscript is now provided through a link to a GitHub repository on page 7 in the Methods section. Upon acceptance, this repository will be updated accordingly. Raw and processed data have been made publically available. The following has been added: "All code used in this study is given in a toolbox and is made publicly available and maintained as a GitHub repository (https://github.com/domdunstan/NerualMassModellingToolbox). Processed data are publicly available on https://osf.io/f2vya/. Raw EEG data can be accessed via text files within the GitHub repository".

Reviewer 1

Major comments

The results show how different objective functions recover different parameter sets, yet the authors claim that different objective functions recover different dynamics. I am aware that changing parameters changes the model behaviour. However, such changes in behaviour are often minor. If the authors wish to state that the different objective functions recover different dynamics, they need to substantiate these claims with additional simulation (could be given in an appendix).

Figure 2 provides exemplar dynamics simulated from optimal parameters recovered from the 4 different algorithms (i.e. using the 4 different objective functions: SOEA20, SOEA45, MOEA20 and MOEA45). The two subfigures containing time series for these simulations (Figure 2a and f) demonstrate both minor and major changes in model behaviour, resulting directly from the use of the different objective functions. For example, unlike the other algorithms, the SOEA20 leads to rhythmic spiking dynamics for subject 13 (light blue trace, Figure 2a). This major difference is apparent in the time series, but is also quantified in differences in the wHVG degree distribution and the amplitude distribution (Figure 2d and e). Notably, it isn't so apparent in the PSD distribution. Figure 2 shows more subtle (minor) differences exist between the SOEA45, MOEA20 and MOEA45, when we look at the time series. However, note, for example, the simulations using parameters recovered from the SOEA45 and MOEA20 algorithms (i.e. the dark blue and purple traces in Figure 2a, b, c, d and e). Although any differences in the time series appear minor, the wHVG degree differences are more striking (Figure 2d), with the wHVG degree distribution of the MOEA20 being closer to the data (black trace). Figure 3 explores this further, demonstrating how apparently minor differences in dynamics between the SOEA45 and the MOEA20 can produce the altered shape of the waveform generated as a result of each algorithm. A practical way to quantify the difference in dynamics is to use the objective scores, which describe how much a simulation deviates from the data. The variability in these scores across all data is given in Figure 4. Supplemental Figure 2 shows across many simulations from all control data analysed, the resulting spectra, wHVG and amplitude distributions. Here, it can be seen the different algorithms give rise to differences in these properties, compared to the data. Additionally, in Supplemental Figure 5, we quantify other properties of the dynamics. In particular, the Hurst exponent is shown to largely differ between the SOEA20 and the other algorithms.

To further clarify these results, on page 14 in the Results section we have added text to explain the changes in dynamics can be major (such as the spikes seen for SOEA20 in Figure 2a) or minor (such as the difference in waveforms seen in Figure 3), as follows: "These changes in dynamics can be major (such as the SOEA20 producing spikes, as shown in Figure 2) or more subtle (such as the difference in waveform between the MOEA20 and SOEA45 encapsulated by the wHVG, as shown in Figure 3)".

I am not really sure what the take home message is in terms of the comparison of the different objective functions. Is one better than the others? Does it depend on what properties of the EEG signal you are

interested in? Or perhaps, one should be combining all four methods and using only the overlapping regions of parameter space?

Ultimately, the choice of algorithm will depend on which properties of the EEG signal one is interested in capturing. Here, the MOEA20 is the best algorithm to use. The reasons for this are that i) we exclude the SOEA20 due to the spiking limit cycle behaviour it can give rise to (Figure 2), ii) we exclude the SOEA45 due to it giving rise to waveforms of a different shape to the data (Figure 3) and iii) we exclude the MOEA45 as it detracts from the dominant alpha rhythm that is seen in the data (Figure 2). Essentially, the MOEA20 provides the best algorithm to capture the alpha rhythm PSD plus the waxing and waning as well as the shape of the waveform (as shown in Supplemental Figure 4). This is why we take the MOEA20 forward to apply to the focal epilepsy data.

To further clarify this in the manuscript we have added the following text to page 20 in the Results section: "Our results thus far show that the MOEA20 is better than the other algorithms at capturing relevant features of the data. We exclude the SOEA20 due to the spiking limit cycle behaviour it can give rise to (Figure 2); the SOEA45 due to it giving rise to waveforms of a different shape to the data (Figure 3); and the MOEA45 as it detracts from dominant alpha rhythms (Figure 2). Essentially, the MOEA20 provides the best algorithm to capture the alpha rhythm PSD plus the waxing and waning, as well as the shape of the waveform (as shown in Supplemental Figure 4). We therefore focus on the MOEA20 and apply it to the problem of understanding differences that have been observed in the dynamics of resting EEG in people with epilepsy versus healthy controls".

Discussion point on page 24 has been expanded as well: "We consider four different objective functions when comparing model output to data. The choice of objective function to use will ultimately be informed by the properties of the EEG one is interested in recapitulating in the model. For the data we analysed, the MOEA20 provided the best way to recreate the PSD of the alpha rhythm, whilst also simulating realistic characteristics in the temporal domain, such as fluctuating amplitudes and the shape of oscillations. We therefore focused on applying the MOEA20 method".

The authors use the sign of the dominant eigenvalue as a measure of whether the dynamics are linear (negative real part) or non-linear (positive real part). This is incorrect. Non-linear systems can have stable fixed points and, as such, eigenvalues which all have negative real parts. Take a stable spiral, for example, variables oscillate with a decaying amplitude as we approach the fixed point. This is not linear behaviour.

We apologise for this confusion. To clarify our point here, we have amended the text in the Results section on page 17/18. The term "nonlinear dynamics" has been changed to "limit cycle dynamics" (which cannot be studied in the linearized system) and "fixed point dynamics" (which can be considered in both the full nonlinear and linearized system).

The previous Table 2 on page 19 has been adjusted to form Figure 9. Figure 9a gives the previous Table 2, with the heading adjusted to "Percentage noise-driven fixed point". Figure 9b and c give exemplar simulations (in phase space) of noise-driven fixed point dynamics and noise-driven limit cycle dynamics, respectively. The Figure caption has also been updated accordingly.

The results for spike-wave dynamics are interesting but seem somewhat like an afterthought. There are many more interesting questions that could be answered here. For example, how similar/different are the parameters values for SWDs to those for regular oscillations in patients with epilepsy? Ideally, the system should live in a region of parameter space where both types of behavior are possible. Then noise or small variations to the parameter values would lead to switches between the two behaviours. I appreciate that such an exploration maybe outside the scope of this paper, but the authors should at least mention how similar/different this parameter set is to the parameter sets found in the previous parts.

The SWD was included to provide an additional example of the benefits of the MOEA20, as well as an additional use case pertinent for epilepsy – being able to mine models for a particular kind of dynamics will aid the search for underlying mechanisms. The SWD used in this study was taken from a different epilepsy patient than the main cohort, since we didn't have seizure recordings from them. Furthermore, for the SWD

data we used, the mean of the frontal electrodes was used to compare to the model output, since the frontal electrodes exhibited the clearest SWD pattern. This contrasts with the resting state data, whereby the mean of the occipital electrodes was used. For these reasons, we are cautious to directly compare parameters recovered from the SWD and parameters recovered from the resting data. However, we agree this would be of interest to the reader, so we have added the recovered parameters from the SWD to form Supplemental Figure 17. Text in the Results section on page 22 has been adjusted to point to this Figure as follows: "Supplemental Figure 17 shows the distribution of parameters recovered from the SWD using the MOEA20 approach".

We believe comparing recovered parameter sets between resting and pathological data, in a more controlled way, would be an interesting avenue for future research. Page 24 in the Discussion has been extended to comment on how future work could investigate the uses of the global MOEA approach discussed, in exploring the parameter paths between healthy and diseased states: "In future, we believe this approach could help to uncover a repertoire of possible mechanisms by which NMMs can transition from a resting to pathological state, thus providing insight into the mechanisms of epilepsy".

Minor comments

Can you tell which parameters contribute most to the differences in the 2-dimensional components?

The multidimensional scaling method can't provide information about which parameters contribute most to the silhouette scores. However, some intuition towards parameter values driving the differences can be gained from comparing univariate parameter distributions. This is explored in Figure 7, where recovered parameter distributions that were most identifiable (and hence most likely to deviate between algorithms) are shown across each method, with differences in identifiability between the methods greatest for parameter γ_e; the excitatory postsynaptic time constant.

Abbreviations

Abbreviations that have not been used for a significant period of time are now restated. This includes DCM on page 23, NMMs on page 23, and SWDs on page 21.

Axis labels and ranges/values

Parameter labels and ranges have been added to the x-axis on Figure 5, 7 and 11. The y-axis on these figures has also been changed to "normalised frequency", to reflect the frequency of parameter occurrence, given the histogram bin size used. These changes have also been made to Supplemental Figures 10, 11, 12, 13, 14 and 15.

Abstract

"comprise many parameters" has been adjusted to "comprise of many parameters". "constrain model output" has been adjusted to "constrain the model output". "MOEA can be used" has been adjusted to "MOEA could be used".

Author summary

"The" has been removed before "EEG".

Methods

The "exp" function has been adjusted to standard font from italics on page 5 of the Methods section. Wording on line 137 has not been changed.

Results

To give an indication of the time course, the following has been added to the Figure 1 caption: “These time points have a resolution of 3.9ms”.

A comment in the Figure 1 caption has been adjusted to “shows the amplitude of 5 consecutive time points”.

“It” has been changed to “it” on page 10.

Figure 7b has been adjusted to plot the Jensen-Shannon divergence over subjects. Significant differences between groups have been calculated (using a Mann–Whitney U test) and are now indicated in the Figure. The Figure caption has been updated accordingly: “These are shown as violin plots over subjects. P-values were obtained from a Mann-Whitney U test, with Bonferroni correction.” Text on page 16 has also be marginally updated to reflect that the MOEA20 has “significantly” larger JSD for 4 out of the 5 parameters.

Reviewer 2

Major comments

I think, before discussing how to choose the model parameters, it is important to discuss how to choose the model. Because, it is the model structure that determines the global dynamical space, not the parameters themselves. Already, the simple neural models with one excitatory and one inhibitory neuron populations (like the Liley model here) ignore the existence of different types of inhibitory interneurons in neocortex (see for instance Bensaid et al. (2019) COALIA: A Computational Model of Human EEG for Consciousness Research. Front. Syst. Neurosci. 13:59. doi: 10.3389/fnsys.2019.00059).

To address this point, we have added to the Discussion on page 25 the caveat that the results are model dependent and future work will explore the application of the MOEA approach to other neural mass models that incorporate different mechanics, such as those that include multiple types of inhibitory interneurons. We believe the MOEA approach can facilitate comparisons between different NMMs, by quantitatively assessing the importance of, for example, different inhibitory populations. We added text on page 25 as follows: “Any inference made from models of course depends on the choice of model, and several different types of NMMs exist (for example, see [1]). Although we focused on the Liley model here, future work will examine the application of the MOEA approach to other NMMs, such as those that contain different types of inhibitory interneurons [4, 12, 51]. We believe the MOEA proposed could provide a better data-driven quantitative analysis of the importance of including different mechanisms in NMMs.

My understanding is that the model output is the mean membrane potential of the subpopulation of excitatory neurons he(t). This contrasts with many other studies in which the model output is taken to be the summated PSPs, both excitatory and inhibitory, at the level of main cells (typically pyramidal cells in cortical regions). Authors should explain their choice.

The rationale for using the membrane potential of the excitatory population as the model output has been established previously, for example see Liley DT, Cadusch PJ, Dafilis MP, A spatially continuous mean field theory of electrocortical activity, Netw. Comput. Neural Syst. 13 (1) (2002) pp. 67–113. doi:10.1080/net.13.1.67.113. Text has been adjusted on page 5 of the Methods section to state that we are following the established model and therefore take the excitatory membrane potential ($h_e(t)$) as the model output.

It is common for other neural mass models to use the net membrane of the excitatory or pyramidal population. In other neural mass models, the net membrane potential is given by the summation of excitatory and inhibitory PSPs received from excitatory and inhibitory sources. In contrast, for the Liley model used here, the inhibitory population implicitly affects the excitatory postsynaptic membrane potential and hence the net membrane potential of the excitatory population is given by the single term $h_e(t)$.

Additionally, it would be informative to see simulated time series after parameter identification, compared with real signals.

In Figure 2, for two example data subjects, we show a simulated time series after parameter identification from each algorithm compared to data. For the first subject, these simulated time series, and how they differ between algorithms, is further explored in Figure 3. In Figure 4, we then characterise the identified dynamics from each algorithm in terms of the objective scores, which determine how similar the dynamics are to the data. Additionally, Supplemental Figure 2 shows the spectra, wHVG and amplitude distributions after parameter identification from many simulations on all control data subjects.

In line with the previous comment, NMM output is a proxy of the local field potential generated in a population of neurons (i.e. a source) comprising sub- populations of excitatory and inhibitory neurons. Authors compare the model output to scalp EEG data recorded from 2 electrodes (occipital for healthy subjects and frontal for people with epilepsy). They should better argue why such a direct comparison is valid in a context where scalp EEG signals reflect the activity of all sources in the brain depending on the source-electrode distance and the source orientation (in accordance with the EEG forward solution).

Explanation of the EEG data on page 6 in the Methods section has been adjusted to more accurately describe the different data sets used in the work. In particular, for both resting data sets (healthy control group and focal epilepsy group) a single signal was used, calculated from the mean of the occipital electrodes.

In comparing to a single signal, we are only modelling a patch of cortex that is assumed to be directly responsible for the alpha rhythm and are therefore neglecting the activity from other sources. In future work that is extended to multiple sources, an appropriate forward model will be used. Page 7 in the Methods section has been updated as follows: “Note that we compare the model output $h_e(t)$ directly to the EEG because in this study we are neglecting contributions from other sources and therefore do not use a forward model. In future work with multiple sources, an appropriate forward model will need to be used”.

Regarding the results section, I would suggest to test SOEA and MOEA methods on signals simulated by the model for different parameter configurations producing alpha activity. This would provide a ground-truth about parameters to recover and thus a complementary test of the performance of methods.

The Results section has been updated to mention that we have now tested each algorithm on 3 simulated subjects. For each of these subjects, the Euclidean distance across the 5 most identifiable parameters were compared between the ground truth and each recovered parameter set. In each case, we found that the MOEA20 approach was at least as good as any of the other algorithms at recreating the ground truth. The Results section on page 19 has been adjusted as follows: “Additionally, we tested the different algorithms on signals simulated by the model. This allowed the distribution of parameters recovered from the algorithms to be compared to a ground truth value. Supplemental Figure 14 shows, across the same parameters as those considered in Figure 7, the Euclidean distance to the ground truth from 3 simulated time series. Supplemental Figure 14 also shows the histograms of parameter values from one of these simulated time series. Across all 3 simulated time series, compared to the other algorithms, we found that the MOEA20 approach was either insignificant from, or significantly closer to, the original ground truth value used to run the simulated data”. A new Supplemental Figure 14 has been added to display these results.

We have also included a point in the Discussion section on using simulated signals. The following has been added on page 24/25: “In addition, we applied our algorithms to model simulations, in order to understand the relationship of recovered parameters with respect to a ground truth. As described above, the notion of “ground truth” should incorporate large regions of parameter space. That is, as we demonstrated in Supplemental Figure 14, given a choice of model parameters to simulate from, the optimisation algorithms will recover large regions of parameter space that yield equivalent, or quantitatively similar, dynamics. Thus the “ground truth” may not be a single value, rather a non-trivial set, or collection of values in a high dimensional space. It is important to be able to map these regions in order to understand the repertoire of dynamics of neural mass models. The algorithms we provided herein offer the means to do this, and in particular the MOEA20 provides a more accurate map”.

In the discussion, it would be nice to address the following point: can the multi-objective optimization algorithm introduced in the article be used to estimate parameters of a NMM from non-stationary data like sporadic epileptic spikes or evoked potentials, for instance? I imagine that wHVG feature can still be used. How would the other objectives be constructed?

We believe this would be an interesting avenue for future research. Previous work has successfully used the wHVG as a detection tool for epileptic seizure dynamics. Text on page 25 in the Discussion section has been added to describe how the MOEA approach discussed in the manuscript could extend some of this work, as fitting mathematical models to data and using the wHVG as an objective could provide insight into the mechanisms that generate these different dynamics: "The wHVG algorithm has also previously been successfully used to delineate between different types of dynamics, including detecting seizure activity in EEG [53]. We believe that using the wHVG as an objective, and the MOEA to fit mathematical models to data, could be a crucial tool to extend these analyses to better understand the mechanisms responsible for generating different dynamics".

Before interpreting the observations on the average synaptic gain (line 471-487), it would be interesting to a bit more clinical information about the 20 people with epilepsy. It is well known that the key variable in the NMMs is the ratio between excitation and inhibition (ePSP and iPSP). Is the decrease of excitation identified from EEG associated with a value of inhibition similar to that identified in HCs. In other words, is the E/I ratio decreased in EEGs recorded in people with epilepsy? In addition, it is mentioned that one of the epilepsy patients is diagnosed with an absence epilepsy. What about the others? Is the cohort homogenous or heterogenous in term of aetiology? These aspects are important for the understanding and interpretation of the excitation decrease.

Supplemental Figure 14 has been adjusted to additionally include the ratio of excitatory to inhibitory postsynaptic gain (i.e. Γ_e/Γ_i). Due to a similar distribution of the inhibitory postsynaptic gain parameter for controls and focal epilepsy subjects, the decrease in excitation found maps onto a lower excitation/inhibition ratio in focal epilepsy subjects compared to healthy controls. We note these results are comparing activity from each of the cohorts during a resting sate. The Discussion on page 24 has been updated as follows: "Due to small differences found in the average synaptic gain for the inhibitory population, these differences observed in Γ_e map onto a lower resting state excitation/inhibition ratio in FE subjects, compared to control. Interestingly, other work has provided evidence for an increase in this ratio in epilepsy patients during the start of a seizure (for example, see [48])".

The subject displaying the SWD rhythm in Figure 12 was diagnosed with absence epilepsy. This subject is separate from the subjects that the resting focal epilepsy data were obtained from. We have adjusted the EEG data subsection in the Methods on page 6 to clarify these different data sets. The section has been updated as follows: "Three data sets were used in total for this work. The first and the second were subsets of data from a previous study [35], and each consist of 20s epochs of EEG alpha activity recorded during the eyes closed resting state. The first set consisted of 20 healthy adults and the second set consisted of adults diagnosed with focal epilepsy (FE). In the cohort that these were taken from, 56% were left lateralised. Further information can be found in [35] and at https://osf.io/f2vya/".

Minor comments

Abstract: In the last paragraph of the abstract, it reads that different EEG states recoded in healthy controls (resting EEG) and in epileptic patients (alpha rhythm EEG). The author summary say it is resting, into says it is resting. Then section 2.2 talks about alpha… The study compares …. Please say that it is "alpha activity recoded during the eye closed resting state"

This comment in the abstract has been adjusted to the following: "EEG alpha activity recorded during the eyes closed resting state".

Figure 1: numbering of the objectives. Inconsistency with the text (pg8)

Figure 1 has been updated to match the objective numbering defined in the text on page 8.

Figure 2: firstly, why are the base lines of normalized power diagrams different? Secondly, why are the y-axes labels in 2d, 2e, 2i, and 2j “normalized frequency”? Finally, visually it is hard to accept that wHVG criteria improved the fitting. Figure 3 zooms into the data presented in 2a, but I find the match is not very convincing. Can authors propose an improvement in the model or algorithm or improve the match?

The normalised power diagrams have different basslines to allow the reader to better differentiate between the different colours. The captions for Figure 2 and Figure 12 have been updated to state that the normalised power is displayed in this way: “To better differentiate between the signals, the baseline of the normalised power is shown with an offset for each algorithm”.

The legend of Figure 2, Figure 3, Figure 12 and Supplemental Figure 2 have been updated to reflect that the normalised frequency y-axis label is used to represent a density distribution that has been calculated from 100 equally sized bins. For Figure 2, the following has been added: “In each case, these signals show a density approximation of the distribution, calculated from 100 equally sized bins (see Methods)”. The Methods section on page 9 has then been updated as follows: “Throughout this work, to visualise differences between wHVG distributions (and also amplitude distributions), we calculate a density approximation of the distribution using 100 equally sized bins”.

In the model simulations, the wHVG distribution was compared to the data across the full 20s time series. In Figure 3, we show an excerpt from the full time series. This highlights differences between data and model time series when using the different objective functions. It also shows these differences are apparent in the wHVG distribution, even for this small excerpt.

Pg3, line 60: Clarify the sentence “We might therefore expect neural mass postsynaptic potentials (PSPs) to be parameterised using values that are different from the values used to parameterise neuronal PSPs.”

The sentence has been adjusted to “we might therefore expect the parameters of neural mass postsynaptic potentials (PSPs) to have different values to the parameters of neuronal PSPs”. This states that parameter values would not necessarily be the same when modelling different scales of neuronal activity.

why are the y-axes labels 3b “normalized frequency”? Face value or silhouette value? Both terms are used.

Figure 3 legend has been adjusted to explain y-axis label for Figure 3b (see above). Face value was used in the general sense, whereas the silhouette value (or score) was used to indicate similarities between clusters. To avoid confusion, on page 11, both uses of the term “face value” have been replaced by the word “initially”.

The “Results” section could be organized in subsections. It would be easier to follow.

The following subsections have been added to Results: “Comparing model dynamics recovered using different objective functions”; “Comparing parameter values recovered using different objective functions”; “Using MOEAs to explain alpha power shifts in epilepsy” and “Optimising model dynamics to spike-wave discharges”.

Additional changes outside of the reviews’ comments:

We have adjusted the Acknowledgements section to reflect the sad passing of a co-author on the paper. The following has been added: “We would like to dedicate this work to the memory of our dear friend and colleague Professor Ozgur Akman”.

“Dynamic Causal Modelling” has been changed to “Dynamic causal modelling” on page 2 and page 23.

“Single objective EAs” has been changed to “SOEAs” on page 23.

“Multi-objective evolutionary algorithm” has been changed to “MOEA” on page 23.

A comment relating to the usefulness of the wHVG in capturing subtleties in the waveform of the data has been added to the Discussion on page 24 as follows: “Additionally, we found that the wHVG objective can help to capture subtleties in the waveform of the data, which were not captured when using the PSD alone (Figure 3)”.

The parameter representing the standard deviation of the noise perturbation (ξ) has been added to the distributions in Supplemental Figure 14.

The opening sentence in a paragraph on comparing model output to data in different ways on page 25 in the Discussion section has been changed to the following: “The problem of deciding how to compare model output to data is non-trivial”.

Y-axis scale on parameter distributions in Figure 11 and Supplemental Figure 15 have been marginally adjusted. The legend on Figure 10 has been adjusted to state that the inserts show the bottom 20% of the parameter ranges.
